# Women and Girls who Carry Knives: A Systematic Review

**DOI:** 10.1177/15248380251338781

**Published:** 2025-06-03

**Authors:** Isabella Pellien, Jane Wood, Emma Alleyne

**Affiliations:** 1 University of Kent, Canterbury, England

**Keywords:** female offending, knife carrying, knife crime, knife-related crime, peer group associations, self-protection, status, fear, respect, psychological motivators

## Abstract

This systematic review explores knife carrying among women and girls, a behavior often overlooked in research and policy. Following Preferred Reporting Items for Systematic Reviews and Meta-Analyses (PRISMA) guidelines and using the Population, Intervention, Comparison and Outcome (PICO) framework, seven databases were searched in February 2022. Studies were included if they examined female knife carrying or knife-related offenses, using all-female or mixed-gender samples, and published in English since 2000. Thirty-three studies met the inclusion criteria, including quantitative, qualitative, and mixed-method designs. Findings indicate that female knife carrying is often examined within broader weapon carrying research, with prevalence rates ranging from less than 1% to 52%, and influenced by sample type (e.g., gang-involved youth). Key situational factors include peer associations, gang membership, and self-protection, while psychological drivers such as status seeking, respect, and fear induction also emerged. However, most research is U.S.-based, limiting generalizability. This review highlights the need for further research on the unique social and psychological dynamics of female knife carrying to inform female-specific interventions.

## Introduction

Knife carrying—the act of carrying a blade or sharp instrument in public (U.K. Parliament, 1953, 1988)—is increasingly prevalent in British society. While this review focuses on knife carrying, official statistics use various terms. In 2018, U.K. knife crime reached a 9-year high (Office for National Statistics, 2019). By 2006, 6% of 10- to 19-year olds identified as gang members, making them three times more likely to carry knives than non-gang peers (Sharp et al., 2006). By 2009, hospitals saw a surge in youth (aged 16 and under) admitted for stab wounds (Centre for Social Justice, 2009). By March 2018, 285 homicides involved knives or sharp objects (Office for National Statistics, 2018). While improved crime recording explains some of this rise (HM Government, 2018), many cases go undetected, suggesting knife carrying is more widespread than reported.

Recent data show knife crime in the United Kingdom disproportionately affects young people, with victims and perpetrators typically aged 12 to 15 (Crime Survey for England and Wales; Office for National Statistics, 2015). Knives often facilitate other crimes, including robberies (Kershaw et al., 2008) and sexual offenses (Woodworth et al., 2013) and are central to gang-related violence. Government reports link gang activity to a 36% rise in knife crime (HM Government, 2018), which is associated with increased child murders (Kirchmaier & Villa Llera, 2018). Understanding knife carrying, therefore, requires examining its broader contexts, particularly among youth and gangs.

While often associated with males, females also engage in knife carrying, although at lower rates. Until recently, female delinquency has been overlooked and often neglected. However, girls have always been involved in violence and delinquency (Schwartz & Steffensmeier, 2012). Browne et al.’s (2022) review found that females primarily use knives in domestic settings, whereas males use them in community settings. In the United States, violent crime arrests for girls rose 87% between 1980 and 2005 (Federal Bureau of Investigation, 2006). In the United Kingdom, London Metropolitan Police recorded a 52% rise in female-perpetrated knife offenses from 2013 to 2018, totaling 916 offenses in 2018. Given their involvement in knife-related crimes, research must be expanded to include girls and young women. This review aims to address the following research questions:

1. What is the prevalence of knife carrying by women and girls?
2. What are the situational/contextual factors that facilitate knife carrying among women and girls?
3. What are the psychological factors associated with knife carrying among women and girls?

### Brief Note on Terminology

Research specifically examining girls involved in knife carrying is limited, and numerous studies have explored the broader context of weapon carrying, which encompasses the act of carrying knives as well as various other types of weapons (e.g., firearms, mace, etc.). Given this varied terminology, we have opted to use the term *knife carrying*, as defined above. Wherever possible, we use this term throughout for consistency, except when reporting specific research that uses another term in a way that deviates from this definition.

## Methods

### Eligibility Criteria

Following Preferred Reporting Items for Systematic Reviews and Meta-Analyses (PRISMA) guidelines (Liberati et al., 2009), the PICO framework (Schardt et al., 2007) guided inclusion criteria. Studies were included if they examined women’s and girls’ involvement in knife crime, either through all-female samples or mixed-gender samples with relevant findings. British Crime Survey data have established the ages of victims and perpetrators of violent assault to be 12 to 15 years old; however, research on female gang membership spans adolescence to adulthood (Choak, 2018; J. Miller & Brunson, 2000; Nurge, 2003; Shaw & Skywalker, 2017; Vigil, 2008). Thus, we used the same age range (adolescence to adulthood) as an inclusion criterion.

To ensure that outcomes reflect differences between those who carry knives and those who do not, we included studies on delinquent, nondelinquent, at-risk, and general populations across clinical, community, school, and forensic settings. Relevant studies examined female knife offending, motivations for knife carrying, and social/psychological factors associated with knife carrying. We included quantitative, qualitative, and mixed-method studies published in English from 2000 onward, as knife crime and knife culture emerged in British crime discourse at this time (Williams, 2023). This time frame ensures that the findings reflect current psychological and contextual influences on girls’ knife carrying. Studies with varying sample sizes were considered to capture diverse evidence.

### Search Strategy

**Figure 1.**
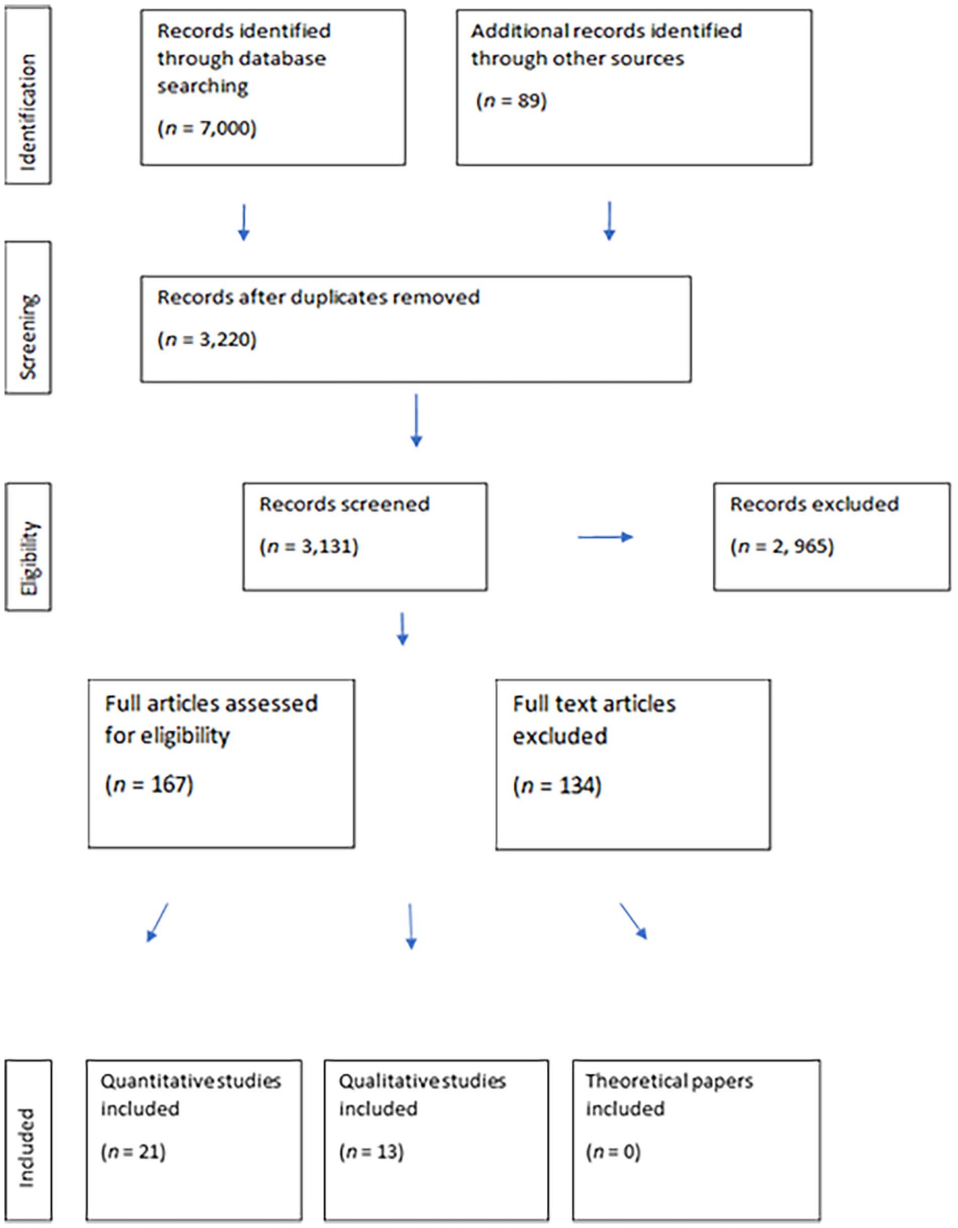
Search process of systematic review adapted from the PRISMA (Moher et al., 2009). *Note.* PRISMA = Preferred Reporting Items for Systematic Reviews and Meta-analyses.

The following electronic databases were searched in February 2022: PsycINFO, PsycArticles, Criminal Justice Abstracts, Wiley, Web of Science, ProQuest, and Academic Search Complete. Search terms fell into two categories: person characteristics (e.g., gender, delinquency, youth) and behavior characteristics (e.g., weapon carrying, knife crime, violence). The final dataset included 33 studies across various regions and methodologies. Figure 1 outlines the search process from paper identification to inclusion in the narrative synthesis

### Data Extraction

Research papers were screened by title and abstract, then further assessed based on study outcomes. Full texts of eligible studies were reviewed by the primary author and evaluated using Kmet et al.’s (2004) quality criteria (Tables 1 and 2). Extracted data included author(s), publication date, study location, aims, design/measures, sample details, comparison group characteristics (e.g., gang vs. non-gang members), and study outcomes (Tables 3–5).

**Table 1.** Quality Assessment for All Included Quantitative Studies as Adapted from Kmet et al. (2004).

Author	1	2	3	4	5	6	7	8	9	10	11	12	13	14	Total Sum	Total possible Sum	Summary Score (%)
Alleyne and Pritchard (2016)	2	2	2	2	N/A	N/A	N/A	2	2	2	2	2	2	2	22	22	100
Aspy et al. (2004)	2	2	2	2	N/A	N/A	N/A	2	2	2	0	0	2	2	18	22	81.82
Auyong et al. (2018)	2	2	2	2	N/A	N/A	N/A	2	0	1	2	2	2	2	19	22	86.36
Barlas and Egan (2006)	2	2	2	2	N/A	N/A	N/A	2	0	2	2	2	2	2	20	22	90.91
Bègue et al. (2016)	2	2	2	2	N/A	N/A	N/A	2	2	2	2	2	2	2	22	22	100
Cotter and Smokowski (2017)	2	1	2	2	N/A	N/A	N/A	2	2	2	2	1	2	2	20	22	90.91
Erickson et al. (2006)	2	2	2	2	N/A	N/A	N/A	2	2	1	0	0	2	2	15	22	68.18
Haynie et al. (2007)	2	2	2	2	N/A	N/A	N/A	2	2	2	0	2	2	2	20	22	90.91
Kerres and Demaray (2003)	2	2	2	2	N/A	N/A	N/A	2	2	2	2	0	2	2	20	22	90.91
Kodjo et al. (2003)	2	2	2	2	N/A	N/A	N/A	2	1	2	0	2	2	2	19	22	86.36
McVie (2010)	2	2	2	2	N/A	N/A	N/A	2	2	2	0	2	2	2	20	22	86.36
Mundt et al. (2017)	2	2	2	2	N/A	N/A	N/A	2	2	2	2	2	2	2	22	22	100
Peterson et al. (2018)	2	2	2	2	N/A	N/A	N/A	2	1	2	2	2	2	1	20	22	90.91
Peterson et al. (2001)	2	2	2	2	N/A	N/A	N/A	2	2	2	0	0	2	2	18	22	81.82
St. Cyr and Decker (2003)	2	1	2	2	N/A	N/A	N/A	0	2	0	0	2	1	2	14	22	63.64
Thornton et al. (2012)	2	2	2	2	N/A	N/A	N/A	2	2	2	0	2	2	2	20	22	90.91
Timchenko et al. (2020)	2	2	2	2	N/A	N/A	N/A	2	1	2	2	0	2	2	19	22	86.36
Wallace (2017)	2	2	2	2	N/A	N/A	N/A	2	2	2	2	2	2	2	22	22	100
Walker-Barnes and Mason (2001)	2	2	2	2	N/A	N/A	N/A	2	0	2	2	0	2	2	18	22	81.82
Watkins and Melde (2018)	2	2	1	2	N/A	N/A	N/A	0	2	1	0	2	2	2	16	22	72.73
Wilcox et al. (2006)	2	2	2	2	N/A	N/A	N/A	2	2	2	0	2	2	2	20	22	90.91

**Table 2.** Quality Assessment for All Included Qualitative Studies as Adapted from Kmet et al. (2004).

Author	1	2	3	4	5	6	7	8	9	10	Total Sum	Total Possible Sum	Summary Score (%)
Choak (2018)	2	2	2	2	2	2	2	2	2	2	20	20	100
Galbavy (2003)	2	2	2	2	2	2	2	0	2	0	16	20	80
Kolb and Palys (2016)	2	2	2	2	2	2	2	0	2	0	16	20	80
Hunt and Joe-Laidler (2001)	2	2	2	1	2	2	2	0	2	0	15	20	75
J. Miller and Brunson (2000)	2	2	2	2	2	2	1	0	2	2	17	20	85
S. Miller et al. (2012)	2	2	2	2	2	2	2	2	2	2	20	20	100
Nurge (2003)	2	1	2	0	2	2	0	0	2	0	11	20	55
Owens et al. (2000)	2	2	2	2	2	2	2	2	2	0	18	20	90
Shaw and Skywalker (2017)	2	1	2	0	0	0	0	0	2	0	7	20	35
Vigil (2008)	1	2	2	1	0	2	2	0	2	0	12	20	60
Vinnakota et al. (2022)	2	2	2	0	2	2	2	2	2	1	17	20	85
Walker-Barnes and Mason (2001)	2	2	2	2	2	2	2	2	2	2	20	20	100
Young (2009)	2	2	2	0	1	2	1	0	2	0	12	20	60

**Table 3.** Summary of Studies Included in Systematic Review (*n* = 33).

Author(s) (year)	Aim of Study	Sample	Comparison Group	Design and Measures	Outcome
Alleyne and Pritchard (2016) The United Kingdom	Investigated psychological and behavioral characteristics in female gang members/female non-gang youths	Girls (*n* = 117) aged 12–18	Gang and non-gang females	Cross-sectional, quantitative, The Youth Survey, Gang Membership, Delinquency, Social Status Scale, Attitude to Formal Authority Scale, Self-Esteem Scale, Hypermasculinity Values Questionnaire, and MSES	Gang members showed higher offense rates, significant differences in sexual activity, and coercion
Aspy et al. (2004) The United States	Investigated the relationships between nine youth assets, demographics, physical fighting, and weapon carrying	1,098 middle school/high school students (*n* = 1,098)	N/A	Quantitative, cross-sectional	Young females were significantly less likely to carry a weapon than young male African Americans and Hispanics were significantly less likely to report carrying a weapon. For weapon carrying, six of nine youth assets were protective
Auyong et al. (2018) The United Kingdom	Examined risk factors for gang involvement	(*n* = 15,445) children examined through adolescence	N/A	Quantitative, longitudinal cohort uses data from the British longitudinal cohort study, ALSPAC, ADHD scale, DAWBA system for depression symptoms, DAWBA system for oppositional defiant disorder symptoms	Girls are less likely to commit crimes than boys. For girls, delinquent peers, oppositional defiance disorder, and social disorganization are associated with an increased likelihood of criminality. Gang-involved girls commit more crimes than girls who are not involved in gangs.
Barlas and Egan (2006) The United Kingdom	Investigated the link between weapon carrying, delinquency, personality, mating effort, and sensational interests	(*n* *=* 121) at-risk adolescents	N/A	Quantitative, JAWS, SRED instrument, SIQ, and MES	Young people carry for intrasexual competition (e.g., portrayal of risk-taking behavior os a means of attracting potential mates). Weapon carrying is used as a power symbol for impressing same-sex rivals. Self-protection and status both cannot explain weapon carrying. Weapon carrying can be attributed to youths’ fascination with aggressive power
Bègue et al. (2016) France	Identified the prevalence of adolescent weapon carrying	(*n* *=* 12,706) youths aged 11–19	N/A	Quantitative cross-sectional, ISRD 2 Questionnaire	Peer delinquency was the strongest predictor of weapon carrying; self-protection was also a factor
Choak (2018) The United Kingdom	Addressed the gap in knowledge among women and road culture	Young women aged 18–25, youth practitioners, criminal justice practitioners	N/A	Qualitative, semi-structured interviews	Key themes: 1. Desire to gain respect by gaining material goods or being feared/known. 2. Lives of female gang members are more diverse than what is accounted for in the literature 3. Women feel like they must do more to gain respect
Cotter and Smokowski (2017) The United States	Identified risk and promotive factors for female aggression	*n* = 2,536 observations at baseline, *n* = 3,055 observations at wave 2, and *n* = 3,580 observations at wave 3 and wave 4 All-female samples	N/A	Quantitative, rural adaption longitudinal study	Internalizing symptoms, delinquent peer associations, and peer pressure were linked with increased female aggression.
Erickson et al. (2006) Canada, the United States, the Netherlands	Describes delinquent girls’ weapon preferences, how often they carried weapons, and risk factors explaining weapon-related violence	(*n* *=* 510) high-risk adolescent females aged 14–17	N/A	Quantitative, cross-sectional	Knives were reported as most frequently used weapon, and mace was the second weapon choice.
Galbavy (2003) The United States	Gathered information on the influence of deviant peers on adolescent criminal behavior and compared male and female offenders on peer influences	(*n* *=* 20) male and female juvenile offenders aged 13–17	N/A	Qualitative, interviews, systematic open-ended questions	Females showed a tendency to self-blame and were less influenced by peers in their delinquent behavior. Females also did not seek peer approval when behaving delinquently.
Haynie et al. (2007) The United States	Examined peer violence/gender composition in friend groups and its link to violence	(*n* *=* 14,044) male and female adolescents	N/A	Quantitative, longitudinal, Add Health Data	Exposure to opposite-sex friends increased the odds of serious violence for females; distinct gender patterns in peer influence emerged (e.g., male influence on girls is greater than female influence on boys)
Kerres and Demaray (2003) The United States	Examined perceptions of social support by students who carry weapons and assessed predictors of carrying a weapon to school	*n* *=* 461 male and female middle school students	N/A	Quantitative, cross-sectional The Child and Adolescent Social Support Scale Health Questionnaire	Boys were more likely to carry weapons; carriers reported lower perceived social support compared to noncarriers
Kodjo et al. (2003) The United States	Looked at the prevalence of weapon carrying among male and female adolescents within a school context.	*n* = 12,105 adolescents and parents spanning from 7th to 12th grade	N/A	Quantitative, cross-sectional Add Health longitudinal data	Weapon carrying was predominantly a male behavior; factors influencing weapon carrying included IPV, witnessing violence, and school connectedness. Ethnicity/gender analyses revealed factors such as neighborhood safety as important for African American females.
Kolb and Palys (2016) The United States	Investigated how gang-affiliated women understood their identities within gangs	*n* = 24 formerly gang-affiliated females ages 18–56	N/A	Qualitative, semi-structured interviews	Women in gangs often adopted masculine traits, challenging traditional femininity within a gendered hierarchical system. Girls engaged in delinquent acts to work toward a respectable status.
Hunt and Joe-Laidler (2001) The United States	Conducted cross-cultural comparisons of how gang members either follow or resist gender expectations	*n* = 141 interviews included with female gang members from three studies	N/A	Qualitative, semi-structured interviews	Explored themes of respect, sexual reputation, and intra-gang dynamics; highlighted factors underlying female-on-female violence.
McVie (2010) The United Kingdom	Provided an account of knife carrying behavior and gang involvement among youths.	*n* *=* 4,300 youths aged 12–17	N/A	Quantitative, longitudinal, ESYTC	Knife carriers were less likely to use their weapon aggressively, suggesting a primary function of self-defense.
J. Miller and Brunson (2000) The United States	Compared descriptions of gang gender composition according to men with descriptions of women.	58 male and female gang members (*n* = 58) aged 12–20	N/A	Qualitative, semi-structured interviews	In all-male gangs, female involvement was seen as inappropriate, whereas in mixed-gender gangs, females actively participated in criminal activity yet faced objectification.
S. Miller et al. (2012) The United States	Analyzed relationships of court-involved girls.	8 adolescent girls (*n* = 8) involved in the juvenile court system.	N/A	Qualitative, content analysis	Revealed themes of distrust in female friendships, preference for positive opposite-sex relationships, and negativity toward boyfriends.
Mundt et al. (2017) The United States	Investigated whether characteristics of adolescent friendship networks influence weapon-related violence.	*n* = 10,482 male and female adolescents (age 12–19)	N/A	Quantitative, National Longitudinal Study of Adolescent Health	Girls who engaged in weapon-related violence were significantly less integrated in friendship networks than girls who did not participate in weapon-related violence.
Nurge (2003) The United States	Examined contexts surrounding female gang involvement.	Community sample of gang/clique involved females (*n* = 58)	N/A	Qualitative interviews	Differentiated between structured gangs and more informal cliques; fights over “disrespect” were common in both settings.
Owens et al. (2000) Australia	Investigated the reasons girls give for using indirect aggression among peers.	54 (*n* *=* 54) 15- to 16-year-old girls, pilot group of 8 (*n* *=* 8) 16-year olds, and 10 (*n* = 8) key teachers	N/A	Qualitative, focus groups and pair interviews	Indirect aggression was driven by the need for acceptance (status), self-protection, jealousy, and competition.
Peterson et al. (2018) The United States	Examined how gender and gang composition (i.e., the sex makeup of the gang) relate to both offending and victimization	Male and female gang youths (*n* = 287)	N/A	Quantitative, cross-sectional	Offending patterns varied by gang sex composition; females in all-female gangs exhibited lower delinquency compared to those in male-majority gangs.
Peterson et al. (2001) The United States	Looked at the sex composition of gangs and their influence on youth delinquency.	369 (*n* *=* 369) male and female gang youths aged 13–15	N/A	Quantitative, cross-sectional	Females in female/independent gangs engaged in fewer personal and property offenses, whereas those in male-majority gangs had higher rates.
Shaw and Skywalker (2017) South Africa	Looked at the roles of women and girls in gangs in Cape Town, SA.	30 female gang members (*n* = 30)	N/A	Qualitative, interviews	Gangs provided a sense of belonging and security; themes of sexual exchange, abuse, and gender-based violence also emerged.
St. Cyr and Decker (2003) The United States	Identified gender differences in perceptions of structural makeup of gangs.	103 (*n* *=* 103) male and female juvenile detainees aged 13–17	N/A	Quantitative, cross-sectional	Males perceived females as less involved in criminal activity, yet females self-reported a significant participation in gang behaviors.
Timchenko et al. (2020) The United States	Added to the existing literature on female gang membership (e.g., experiences of victimization, involvement in violent crime and delinquency, contact with criminal justice system).	*n* = 90,000 adolescent youths	N/A	Quantitative, using longitudinal data, involvement in criminal justice system, gang membership, violent crime/delinquency	Female gang membership was linked to higher arrest rates, increased delinquency, and more victimization experiences.
Thornton et al. (2012) The United Kingdom	Assessed women’s violent and nonviolent offending behaviors	*n* = 344 females aged 18–68	N/A	Quantitative	Women were involved in both violent and nonviolent offending, with nonviolent offenses comprising the largest proportion.
Vigil (2008) The United States	Investigated females affiliated with gangs in East LA	12 girls (*n* *=* 12) aged 12–24	N/A	Qualitative, informal interviews and observations	Female roles extended beyond subservience, with evidence of leadership and aggression driven by jealousy and competition for male attention. Gang girls also function as bait, setting up rival members/luring them to be attacked.
Vinnakota et al. (2022) The United Kingdom	Evaluated knife crime in the United Kingdom from 2011 to 2021	*n* *=* 692 online news reports of knife crime	N/A	Content analysis of online news articles	61.8% of knife crimes were reported in South England; street violence, gang attacks, family issues, and robbery were leading causes; 6.4% of perpetrators were female.
Wallace (2017) The United States	Investigated the perceived popularity of adolescent weapon carriers compared to those who carry and use weapons in violent acts/threatened violence	*n* = 7,106 adolescents in wave 1, *N* = 2,729 adolescents in wave 2 (age 13–17)	N/A	Quantitative, cross-sectional, National longitudinal study of Add Health	Weapon carrying/use was more common among males; among females, differences in social networks between carriers and noncarriers were evident.
Walker-Barnes and Mason (2001) The United States	Explored girls’ perceptions of risk factors for female gang involvement	*n* = 31 female students	N/A	Mixed-methods, semi-structured interviews	Gang membership was seen as a route to identity formation and respect, with joining gangs perceived as beneficial for status/respect.
Watkins and Melde (2018) The United States	Looked at weapon carrying prevalence among male and female gang members	13,097 (*n* = 13,097) male and female gang members	N/A	Quantitative, longitudinal	Prevalence of weapon carrying and risky weapon carrying were higher for gang girls than non-gang girls.
Wilcox et al. (2006) The United States	Investigated relationships between weapon carrying and experiences of crime in a school context	4,000 (*n* *=* 4,000) students	N/A	Quantitative, longitudinal	Weapon carrying was linked to increased fear, risk, perpetration, and victimization; the defensive weapon carrying hypothesis was not supported.
Young (2009) The United Kingdom	Examined female gang members and their involvement with gangs/weapon use	25 (*n* *=* 25) young women aged 14–20	N/A	Qualitative, semi-structured interviews	Girls displayed increased aggression, relied on male protection, and sought peer approval primarily from other females.

*Note.* JAWS = Juvenile attitudes toward weapon scale; SRED = self-report early delinquency; SIQ = sensational interests’ questionnaire; MES = mating effort scale; ESYTC = Edinburgh Study of Youth Transitions and Crime.

**Table 4.** Summary of Findings.

Key Themes	Findings
Prevalence of knife carrying	Female knife carrying is less prevalent than male knife carrying, but it is still occurring. Prevalence rates are between <1% (Begue et al., 2016) and 52% (Erickson et al., 2006). Research largely focuses on weapon carrying broadly, therefore, female knife carrying is overlooked as a distinct behavior.
Situational/contextual factors	Knife carrying among females is linked to gang affiliation (Haynie et al., 2007; Miller & Brunson, 2000; Peterson et al., 2001, 2018), peer influences (Barlas & Egan, 2006; Cotter & Smokowski, 2017; Mundt et al., 2017; Wallace, 2017), and self-protection motives (Begue et al., 2016; Erickson et al., 2006; McVie, 2010; Wilcox et al., 2006; Young, 2009). However, self-protection as a direct cause remains inconclusive due to cross-sectional design limitations.
Psychological factors	Status, respect, and fear (Choak, 2018; Kolb & Palys; 2016; Laidler & Hunt, 2001; Miller & Brunson,2000; Walker-Barnes et al., 2001) are key motivators. Females in male-majority or mixed-gender peer groups often engage in knife carrying to gain social capital (Haynie et al., 2007; Miller & Brunson, 2000; Peterson et al., 2001, 2018).

**Table 5.** Implications for Practice, Policy and Research.

Implication Type	Recommendations
Practice	Female-specific prevention and intervention strategies are needed. Given the role of peer groups and gang involvement, policies should incorporate community-based intervention programs that engage at-risk girls in mentorship, education, and employment opportunities.
Policy	Most policies addressing youth violence are *male-focused*. There is a need for female-specific policies that acknowledge the distinct social, psychological, and situational factors contributing to knife carrying among women and girls. Research shows that suspension is linked to weapon carrying among females (Kodjo et al., 2003). Schools should implement restorative justice practices and intervention programs rather than punitive measures that may push at-risk girls further toward criminal behavior.
Research	Future studies should explore non-offending females who carry knives, and more U.K.-based research is needed to contextualize findings beyond the U.S. perspective. Additionally, conflicting findings with regard to race suggest that it could play a factor in influencing knife carrying among females. Future research should investigate how race may shape motivations for knife carrying. The neighborhood safety concerns found among African American females (Kodjo et al., 2003) suggest that some racial/ethnic groups may be more likely to carry weapons in response to perceived environmental threats. Future studies should explore how perceptions of safety, policing, and community violence exposure contribute to knife carrying behavior.

## Results

### Description of Studies

The review synthesized findings from 33 studies—21 quantitative (primarily cross-sectional), 13 qualitative, and 1 mixed-method study. Samples included both all-female (*n* = 13) and mixed-gender (*n* = 20) groups from the United States (*n* = 22), United Kingdom (*n* = 8), and other countries.

## Key Findings

### What is the Prevalence of Knife carrying by Women and Girls?

Most research reviewed for this section focused on weapon carrying as a broader term encompassing knife carrying as well as various other types of weapons. Following the appropriate screening process, a total of nine studies reported a prevalence rate of knife carrying by women. What follows is a brief overview of evidence, which estimates the prevalence of knife carrying among women.

Knife carrying may not always result in a violent offense, which means that prevalence trends of the increase in such behavior might not be representative. A content analysis of U.K. online news portals reported the total number of perpetrators involved in knife crime from January 2011 to December 2021. For this study, the term “knife crime” was defined as using a knife in the commission of a crime. Of the 879 participants, just 6.4% (*n* = 56) were females (Vinnakota et al., 2022). The percentage of female involvement in knife crime, while relatively low, is noteworthy as it represents a portion of girls engaging in such criminal behavior.

Another U.K. study with 344 participants reported high rates of general violent offending (e.g., used a weapon on someone, threatened someone with a weapon) among women (50%–78.3%) (Thornton et al., 2012). However, it is important to note that the study’s sample size was relatively small. Watkins and Melde (2018) found that while non-gang girls reported weapon use at 4% (wave 1) and 2% (wave 2), gang girls reported rates of 25% to 29%. For risky weapon carrying, the prevalence rate for non-gang girls was at 5% (wave 1) and 3% (wave 2) and 29% (wave 1) and 25% (wave 2) for gang girls (Watkins & Melde, 2018). These findings demonstrate that females are engaging in knife carrying behavior, and it is evident that contextual factors, such as gang membership, have a significant influence.

A French study (*n* = 12,706) showed that only 0.9% of participants indicated that they carried a weapon in the last 12 months. Of the total sample (*n* *=* 12,706), only 0.3% of females indicated they had carried a weapon in the last 12 months compared to 1.6% of males (Bègue et al., 2016). Haynie et al. (2007) observed 3.5% of females’ involvement in serious violence (e.g., robbery, pulling a knife/gun on someone, shooting/stabbing someone) in wave 2.

In Haynie et al.’ s (2007) study, using a weapon/knife to get something from someone (e.g., robbery), pulling a knife/gun on someone, or shooting/stabbing someone was measured by wave 2 in-home survey. Among a sample of 14,044 adolescents, 7% reported engaging in one or more of these acts (4% involved in one act and 3% involved in more than one act) (Haynie et al., 2007). Descriptive statistics revealed females to be less involved in serious violent acts when compared to male respondents (Haynie et al., 2007). Overall, only 3.5% of females reported involvement in serious violence (e.g., robbery, pulling a knife/gun on someone, shooting/stabbing someone) in wave 2 (Haynie et al., 2007).

A U.K.-based study conducted by Young (2009) interviewed 49 young people at risk of offending or who had already offended. Within this sample were 25 young women identified by youth offending team managers as having been involved in group offending. Of this, two women admitted to having engaged in knife carrying (Young, 2009). Additionally, knife carrying was identified as being associated with young men more (Young, 2009). In another U.K. study, 10% of females reported involvement in knife carrying (e.g., carrying a “bladed” weapon) (Barlas & Egan, 2006). Moreover, 3% of 59 females reported using a weapon to threaten and 3% reported using a weapon to injure (Barlas & Egan, 2006). Like the findings of other studies (Bègue et al., 2016; Haynie et al., 2007; Young, 2009) males were significantly more likely to carry weapons than females (Barlas & Egan, 2006).

Other school-based studies (Aspy et al., 2004; Erickson et al., 2006; Kodjo et al., 2003; Mundt et al., 2017; Vıgıl, 2016; Wallace, 2017) further confirmed lower female rates—with some geographic variations noted (Erickson et al., 2006). While Wallace (2017) did not provide an exact percentage, the study noted that weapon use was more common among males. Mundt et al. (2017) reported that 26.2% of girls (11 out of a sample of 10,482, where 54% were female) carried a weapon such as a knife, gun, or club, with girls overall showing significantly less involvement than boys. Erickson et al. (2006) found that knife carrying was the most frequently reported form of weapon carrying, with about 25% of girls doing so overall; however, this varied by location. In Toronto, 52% of 510 females reported knife carrying, and school-based weapon carrying rates ranged from 31.6% in Toronto to 12% in Montreal, with Philadelphia reporting that 25% of girls always or often carried a weapon, compared to 5.4% in Montreal and 13.3% in Amsterdam. Kodjo et al. (2003) further confirmed that males were significantly more likely than females to carry weapons, while Aspy et al. (2004) found that female youth were more likely to report not carrying any weapon. Overall, prevalence rates vary widely, with lower rates under certain conditions (e.g., larger sample sizes) and higher rates under other conditions, notably those studies with high-risk and offending samples. In sum, we can conclude that the most rigorous studies combined indicate prevalence rates of female knife carrying ranging from less than 1% to as high as 52%. Nevertheless, further research is needed to unravel the complex interplay between the situational contexts and psychological drivers that underpin knife carrying.

### What are the Situational/Contextual Factors That Facilitate Knife carrying among Women and Girls?

Knife carrying is strongly linked to serious group-related offending and often emerges in youths with prior victimization, with many citing self-protection as a motive (Young, 2009). Knife carrying is embedded in a risky lifestyle, including violent and nonviolent offending and associating with delinquent peer groups. To grasp an understanding of the contexts knife carrying occurs, key elements to consider include peer group associations, gang involvement, and self-protection. In the following section, a cumulative total of 25 studies presents an overview of situational/contextual factors that may facilitate knife carrying.

Much of the research available is based on male perceptions of females holding/carrying weapons for male gang counterparts, with a small amount of work using exclusively female samples. Although traditional roles in gangs have cast females as subordinate (J. Miller & Brunson, 2000; Kolb & Palys, 2016; Shaw & Skywalker, 2017), recent research shows increasing female participation in violent crime. For example, U.S. longitudinal data observed that females were more involved in criminal activities attributable to gang membership when compared to males (Watkins & Melde, 2018), and U.K. studies have found that gang-involved girls commit more crime than those not in gangs (Alleyne & Pritchard, 2016; Auyong et al., 2018). Qualitative accounts suggest that some females may even exhibit violence comparable to or exceeding that of males as a means to prove themselves (Choak, 2018).

The above findings suggest that association with delinquent peers links to higher levels of delinquency and offending among those females involved with either male-majority or mixed-peer groups (Haynie et al., 2007; J. Miller & Brunson, 2000; Peterson et al., 2001, 2018). Cross-sectional design (Peterson et al., 2001, 2018) as well as longitudinal work (Haynie et al., 2007) in the United States identified significant differences in gender composition within gangs and peer groups and delinquency/offending. Findings from Peterson et al. (2001) revealed that girls in all-female and majority-female gangs had the lowest rates of delinquency (e.g., stealing, carrying weapons, committing robberies, attacking others, shooting at someone), meanwhile, girls in mixed-gender gangs had higher rates of delinquency. While this study focused on a young sample (ages 13–15), another U.S. study utilizing an older sample maintained similar patterns in terms of the impact of sex structure on males’ and females’ experiences within the gang (J. Miller & Brunson, 2000), suggesting that gender composition influences experiences even in adult female members. Multivariate analysis revealed that among females, exposure to opposite-sex friends increased their involvement in serious violence (Haynie et al., 2007). More recent work conducted within-sex analyses and found that the level of delinquency among females varied depending on the gender composition of their gangs. Specifically, females within male-majority gangs exhibited the highest levels of delinquency, followed by females in sex-balanced gangs, with females in all-female gangs exhibiting the lowest levels of delinquency (Peterson et al., 2018). Interestingly, research by Timchenko et al. (2020) found that although gang membership among females is associated with violent behavior (e.g., whether participants had pulled a knife/gun on someone, whether they had stabbed someone, how many times they had used a weapon to attain something, and how often they had used a weapon in a fight) the effect sizes are small, suggesting that the strength of this link remains open to interpretation.

S. Miller et al.’s (2012) U.S.-based qualitative work revealed contradictory findings in which delinquent girls did not engage in delinquency with male peers. However, the small sample size (*n* = 8) may limit its generalizability. S. Miller et al.’s (2012) qualitative work investigated the specific context of female delinquency when surrounded by male peers, and the findings may reflect those that may be unique and atypical to those participants. The qualitative approach of S. Miller et al. (2012) identified several situational factors that were overlooked in quantitative work. That is, girls’ relationships—particularly feelings of distrust toward same-gender peers, the appeal of non-romantic friendships with older males, and the challenges present in romantic relationships—shaped their experiences of delinquency by influencing who they spent time with, the environments they felt safe in, and the kinds of behaviors they engaged in to maintain or protect those relationships. For example, some girls described shoplifting or skipping school with peers as a way to bond, while others maintained ties with older male groups for protection and emotional support, even if it meant being exposed to risky settings or behaviors. The study also brought attention to the complex balance between personal agency and peer influences, something that quantitative studies missed. For many of the girls, delinquent behavior was not only just about risk, but also offered a sense of fun, belonging, or safety. These insights are especially valuable for shaping future qualitative work focused on relational dynamics and developing more nuanced quantitative measures that reflect how young women experience and explain their behavior. Additionally, participants in quantitative surveys may be susceptible to social desirability bias. The findings posit valuable insights that may not be representative of the broader trends found in the quantitative studies. However, further examination of gang and non-gang-involved women is necessary to grasp a better understanding of whether gang membership is associated with weapon carrying.

Findings on peer dynamics are mixed. While Cotter and Smokowski (2017) and Wallace (2017) reported that gang members using weapons felt more socially connected, other studies found that less popular girls—with fewer friends and less social support—were more likely to carry weapons, possibly to offset feelings of isolation or vulnerability (Kerres & Demaray, 2003; Mundt et al., 2017).

Findings from Wallace (2017) suggest that gang members who used weapons in situations of violence perceived themselves as more socially connected and popular. In a separate study, association with delinquent peers and peer pressure emerged as salient risk factors for female aggression (Cotter & Smokowski, 2017). However, Kerres and Demaray (2003) and Mundt et al. (2017) found that less popular girls—with fewer friends and less social support—were more likely to carry weapons. Mundt et al. (2017) corroborated these findings, observing that girls who were less popular were more likely to engage in weapon-related crime and weapon carrying as young women. These findings might also suggest that girls with fewer friends are at a higher risk of victimization/fear of victimization, which may in turn lead to carry a weapon as a means of self-defense. Equally, it could be that girls who are inclined to carry and potentially use weapons are not particularly attractive prospects for friendships.

Regarding self-protection, several studies suggest that weapon carrying serves as a defensive mechanism (Bègue et al., 2016; Erickson et al., 2006; McVie, 2010; Young, 2009). For instance, McVie (2010) noted that knife carriers were less likely to use their weapon against someone, indicating a lack of intent to harm. Though Erickson et al.’s (2006) research did not directly examine weapon carry as a self-defense method, mace was preferred as a second weapon choice, suggesting that self-protection was an important factor for weapon carrying for participants in this study. This was suggestive that self-protection was an important factor for weapon carrying. Other research (Barlas & Egan, 2006; Wilcox et al., 2006) did not find strong support for self-protection as the primary motive.

Additional factors—such as substance use, school suspension, perpetration of physical violence, and being African American or Latina (Kodjo et al., 2003)—also influence weapon carrying among females. Further ethnicity and gender analyses revealed that neighborhood safety was an important factor for African American females (Kodjo et al., 2003). Contrary to Kodjo et al.’s (2003) findings, Aspy et al.’s (2004) work revealed African Americans and Hispanics were significantly less likely to report carrying a weapon. Aspy et al. (2004) did use self-report measures, so it is plausible that limitations may have derived from the need to provide socially acceptable responses, thereby influencing the validity of responses (e.g., underreporting of weapon carrying).

In sum, peer group associations emerge as a key theme in female knife carrying. Research indicates that females involved in male-majority or mixed-peer groups tend to engage in higher levels of criminal activity (Haynie et al., 2007; J. Miller & Brunson, 2000; Peterson et al., 2001, 2018), with peer influences also facilitating weapon carrying in school-based contexts (Barlas & Egan, 2006; Cotter & Smokowski, 2017; Mundt et al., 2017; Wallace, 2017). Some studies suggest that knives are carried for protection, as indicated by the absence of intent to harm (McVie, 2010; Young, 2009); however, cross-sectional designs (Bègue et al., 2016) and a lack of direct examination of weapon carrying as self-defense (Erickson et al., 2006) limit definitive conclusions. Findings on race are mixed: Kodjo et al. (2003) found race as a factor in female weapon carrying, while Aspy et al. (2004) reported that African American and Latina youths were less likely to report carrying weapons. The literature predominantly focuses on school contexts rather than exclusively on female gang youths (Barlas & Egan, 2006; Cotter & Smokowski, 2017; Mundt et al., 2017; Wallace, 2017), and sampling issues—such as truancy—may omit high-risk adolescents. Moreover, with most studies being U.S.-based, generalizability to other settings is limited. Ultimately, a deeper exploration of the psychological processes underlying female knife carrying is essential for understanding its role in violent behavior.

### What are the Psychological Factors Associated with Knife carrying among Women and Girls?

A thorough examination of psychological factors related to female knife carrying can lead to a deeper understanding of the issue and is imperative for female-specific prevention and intervention efforts. The following section presents a review of 10 studies examining psychological factors associated with knife carrying in women and girls.

Research consistently identifies status, respect, reputation, competition, and forms of indirect aggression (e.g., gossip, backbiting, social exclusion) as key motivators (Choak, 2018; J. Miller & Brunson, 2000; Kolb & Palys, 2016; Laidler & Hunt, 2001; Owens et al., 2000; S. Miller et al., 2012; Vigil, 2008; Walker-Barnes & Mason, 2001; Young, 2009). In male-majority or mixed-gender groups, females actively seek status and respect—often attaining it through material displays and instilling fear (Choak, 2018; Walker-Barnes & Mason, 2001). J. Miller and Brunson’s (2000) U.S. study showed that even though some gang members viewed female participation as “inappropriate,” those who conformed to male behaviors gained respect and even “honorary male” status. Another U.S. study also revealed that fear was a common theme across items and participants expressed that a way to obtain respect was through fear (Walker-Barnes & Mason, 2001). The theme of fear was also present in explanations of how being in a gang can make one feel important (Walker-Barnes & Mason, 2001).

Hunt and Joe-Laidler (2001) revealed themes of “respectability” and reputation in the analysis for both interactions with males and interactions with females. Females in gangs worked hard to show they deserved equal status to their “homeboys” and put in work to ascend the hierarchical system in gangs. One girl, Alma, was considered a “homegirl” because she worked hard to prove herself to male counterparts (Kolb & Palys, 2016). Among other themes that emerged from Hunt and Joe-Laidler’s (2001) study was the notion of “respectability” in interactions with other girls, which they expanded on as sexual reputation. The girls described “respectability” as synonymous with restraint (e.g., avoiding promiscuity, staying in control while partying, and distancing themselves from girls seen as “slutty” or “out of control”). These norms were actively policed by other girls, often through gossip, exclusion, or confrontation. Upholding a respectable image was seen as essential to gaining and maintaining status, avoiding harassment, and being taken seriously within the group. While knife carrying was not explicitly mentioned in the study, violence was frequently described as a way to defend one’s honor when that reputation was threatened. One girl, for example, reported being “almost stabbed” during a fight triggered by perceived sexual betrayal. Such accounts suggest that weapon-related violence, including knife use, may be linked to efforts to protect status and femininity in peer conflicts, even if not central to the study’s focus. Females also reported a reason for female-on-female violence as promiscuity or “slutty” behavior (Laidler & Hunt, 2001). Interpersonal criticism (self-policing, complaining, etc.) was ever present among gang girls and was suggestive of the value of the male gaze to female gang members in this study (Laidler & Hunt, 2001). Girls who were viewed as “slutty” or overly flirtatious—particularly those who engaged with multiple male gang members—were seen as tainting the reputation of the group. This was not just about individual judgment but about the social consequences that came with being associated with such behavior. As one participant explained, her group broke away from their former set because “the girls were just real slutty, real hos,” noting that their behavior “makes everybody look bad” (Laidler & Hunt, 2001). In some cases, this reputational damage led to physical violence. For example, one respondent reported attacking another girl with a bat after walking in on her with her boyfriend. These reactions highlight how some female participants enforced group-defined sexual norms through violence, positioning themselves as gatekeepers of “respectability” in peer settings (Laidler & Hunt, 2001). In this context, violence manifested as a means of disciplining behavior that was seen as disloyal or inappropriate, reinforcing group norms within a male-dominated street culture. Owens et al. (2000) and Vigil (2008) further noted that competition over the opposite sex is a significant form of inter-female aggression observed in both adolescent and adult samples.

S. Miller et al. (2012) highlighted social aggression, including backbiting (speaking negatively about someone in their absence), social exclusion, and distrust of same-gender peers. In contrast, Young (2009) emphasized the importance of female approval, a finding that differs from Laidler and Hunt’s (2001) observations of adherence to patriarchal norms. Interestingly, Galbavy (2003) found that female delinquency appeared independent of peer influences, suggesting variability in these dynamics. However, this study may be limited due to the participants being incarcerated. Different themes could have emerged in a sample of adolescents who were not incarcerated. In sum, the current research points to a range of negative behaviors and indirect aggression linked to females, with nuanced findings on the role of female approval.

Overall, status, respect, and fear emerge as dominant psychological drivers for knife carrying among females (Choak, 2018; J. Miller & Brunson, 2000; Laidler and Hunt, 2001; Walker-Barnes & Mason, 2001). However, most studies focus on specific populations, such as gang-involved or incarcerated individuals, rely on self-report data, and are predominantly U.S.-based—limiting the generalizability of these findings. Further research comparing female knife carrying with broader female offending patterns is needed to clarify these distinct motivational factors.

## Discussion

This review synthesized findings from 33 studies categorized by prevalence (9 studies), situational/contextual factors (25 studies), and psychological motivators (10 studies). Although most research treats knife carrying within the broader context of weapon carrying (Aspy et al., 2004; Barlas & Egan, 2006; Bègue et al., 2016; Erickson et al., 2006; Haynie et al., 2007; Kodjo et al., 2003; Mundt et al., 2017; Thornton et al., 2012; Wallace, 2017; Watkins & Melde, 2018), our findings reveal notable variability in female knife carrying rates, influenced by factors such as sample size, risk levels, and geographical context. Key situational factors include involvement in male-dominated or mixed-gender peer groups and gangs (Haynie et al., 2007; J. Miller & Brunson, 2000; Peterson et al., 2001, 2018)—suggesting that a desire to prove oneself to male counterparts and peer influences play central roles. While some studies link knife carrying to self-protection (Bègue et al., 2016; Erickson et al., 2006; McVie, 2010; Wilcox et al., 2006; Young, 2009), the evidence is inconclusive due to cross-sectional designs. Psychologically, the pursuit of status and respect (Choak, 2018; J. Miller & Brunson, 2000; Laidler & Hunt, 2001; Walker-Barnes & Mason, 2001)—and the use of fear as a means to achieve these (Choak, 2018; Walker-Barnes & Mason, 2001)—emerge as prominent motivators. However, these findings mainly reflect offender populations, indicating the need for further research among non-offending samples. These findings may also have implications for understanding other forms of violence (e.g., acid attacks, gun violence). While differing in tactics, other forms of violence may share similar themes (e.g., status-seeking, retaliation, gendered expressions of power). Comparative research across weapon types could reveal whether similar psychological and contextual motivators underpin these behaviors, further informing broader violence prevention efforts.

Although this review did not adopt a single overarching theoretical model, the findings can be interpreted through the lens of Social Role Theory, which posits that gendered behavior arises from culturally defined social roles (Eagly, 1987). This is evident in the ways girls in male-dominated peer groups may adopt behaviors—such as knife carrying—that align with dominant masculine norms to gain respect and status. Additionally, insights from Relational–Cultural Theory (J. B. Miller, 1976) help explain the relational dynamics underpinning knife carrying. Namely, insights from Relational–Cultural Theory (J. B. Miller, 1976) emphasize the role of relationships (particularly for women and girls) in human development. When these relational needs are threatened—through experiences of social exclusion, disconnection, or peer rejection—individuals may engage in maladaptive strategies to regain a sense of control, connection, or protection. In the context of this review, girls with fewer friends or lower social status were more likely to carry weapons, potentially as a response to feeling vulnerable or at risk of victimization. Knife carrying may thus serve as a means of navigating power imbalances, gaining perceived social protection, or reestablishing belonging in peer networks where dominance and toughness are valued. This framework provides a compelling lens for understanding the emotional and relational functions of knife carrying beyond purely instrumental or fear-based explanations.

These frameworks point to the importance of considering both social role expectations and the impact of disruption in relationships when trying to understand why some girls engage in knife carrying behavior. Rather than viewing this behavior solely through a punitive lens, the findings suggest that more relationally focused and identity-sensitive approaches are needed—ones that consider how girls navigate belonging, power, and connection in their social worlds.

## Conclusion and Diversity Considerations

A lot of the existing research looks at weapon carrying in broad terms, which means female knife carrying often gets overlooked as a unique behavior. Factors like race and neighborhood safety play a critical role, yet this behavior remains understudied. Addressing this gap requires research that explores the social, cultural, and environmental influences on female knife carrying. Future studies would benefit from prioritizing longitudinal studies that explore knife carrying trajectories among non-offending females as well as investigate the role of trauma exposure, social exclusion, and cultural norms in driving such motivations. Furthermore, future research should employ detailed ethnic and gender analyses to understand the combined effects of gender, ethnicity, and social background on the reasons women and girls engage in knife carrying. For instance, experiences of systemic racism, cultural norms surrounding femininity, community-level violence, and distrust in law enforcement may differentially influence the perceived need for self-protection or engagement in group-related violence. Additionally, little research has explored how knife carrying behaviors manifest among transgender, nonbinary, or gender-nonconforming individuals, representing a significant gap in understanding knife carrying beyond binary gender norms. Intersectional analyses are critical to developing nuanced, culturally competent, and gender-affirming prevention and intervention strategies.

While we previously highlighted the importance of intersectional research, we also recognize the need to align this with practical intervention efforts. Most youth violence policies and programs are still heavily male-focused, which risks overlooking the specific social, psychological, and situational factors influencing knife carrying among girls. Female-specific prevention and intervention strategies are urgently needed—particularly ones that acknowledge the role of peer groups, gang affiliation, and gendered pressures around status, protection, and respectability.

Community-based interventions that engage at-risk girls through mentorship, education, and employment opportunities could be especially impactful. Punitive measures like suspension may unintentionally push vulnerable girls closer to street-involved or criminal behavior. Instead, schools should consider implementing restorative justice and trauma-informed responses that create space for early intervention.

There’s also a need for more research into girls who carry knives but do not offend, as well as a greater focus on the U.K. context, where much of the existing literature is U.S.-based. Racial identity may further shape motivations for knife carrying, and conflicting findings in this area point to a need for deeper exploration. For instance, Kodjo et al. (2003) found that African American girls were more likely to carry weapons due to concerns about neighborhood safety, which raises questions about how policing practices, community violence exposure, and perceived threat contribute to weapon carrying among specific racial and ethnic groups.

Most of the studies included in this review were conducted in the United States or United Kingdom, which limits the extent to which findings can be generalized to other cultural or geographical contexts. In countries where gender norms, weapon laws, gang dynamics, or levels of community violence differ, the motivations for knife carrying may look very different. For example, in some regions, knife carrying may be more closely linked to political unrest, familial expectations, or community-based protection roles. Future research should explore how these behaviors manifest globally, paying particular attention to regions currently underrepresented in the literature, such as Sub-Saharan Africa, Latin America, and parts of Asia and the Middle East. Doing so would help identify both shared and culturally specific risk factors and better inform the development of interventions. Ultimately, culturally competent, gender-responsive, and context-aware strategies—both in research and in practice—are essential if we want to develop interventions that resonate with the young people most at risk.
